# Retrograde endosonography for diagnosing imaging-occult cancer at the head of the pancreas in a patient with distal gastrectomy and Roux-en-Y reconstruction

**DOI:** 10.1016/j.vgie.2024.03.018

**Published:** 2024-04-04

**Authors:** Michael Lajin, Helen Sohn, Igor Medic, Octavio Armas, Kian Bagheri

**Affiliations:** Sharp HealthCare, San Diego, California

## Introduction

EUS is more sensitive than cross-sectional imaging in detecting early pancreatic cancer.^1^ Altered anatomies present a challenge to imaging the head of the pancreas using EUS due to the inability of the echoendoscope to reach the duodenum.

The EUS-directed transgastric ERCP (EDGE) technique enables the performance of a biopsy of a tumor at the head of the pancreas in a patient with gastric bypass anatomy.^2^ However, distal gastrectomy with Roux-en-Y reconstruction is more challenging due to the absence of “an excluded stomach,” which prohibits accessing the duodenum in an antegrade fashion.

## Case

A 73-year-old man, a victim of an explosion that led to a distal gastrectomy many years ago, presented with jaundice and a 20-pound weight loss over the last 2 months. His alkaline phosphatase level was 704 IU/L (normal: 35-129 IU/L), his bilirubin level was 7.3 mg/dL (normal: 0-1.2 mg/dL), and his carbohydrate antigen 19-9 level was 267 U/mL (normal: 0-35 U/mL). Abdominal CT with intravenous contrast showed cholelithiasis and a dilated biliary tree without evidence of choledocholithiasis or neoplasm. MRCP was not possible due to shrapnel. He had a failed enteroscopy-assisted ERCP and was transferred to our hospital.

To verify his anatomy, push enteroscopy revealed a distal gastrectomy, a gastrojejunostomy, and a jejuno-jejunostomy consistent with distal gastrectomy with Roux-en-Y reconstruction.

**Figure 1:**
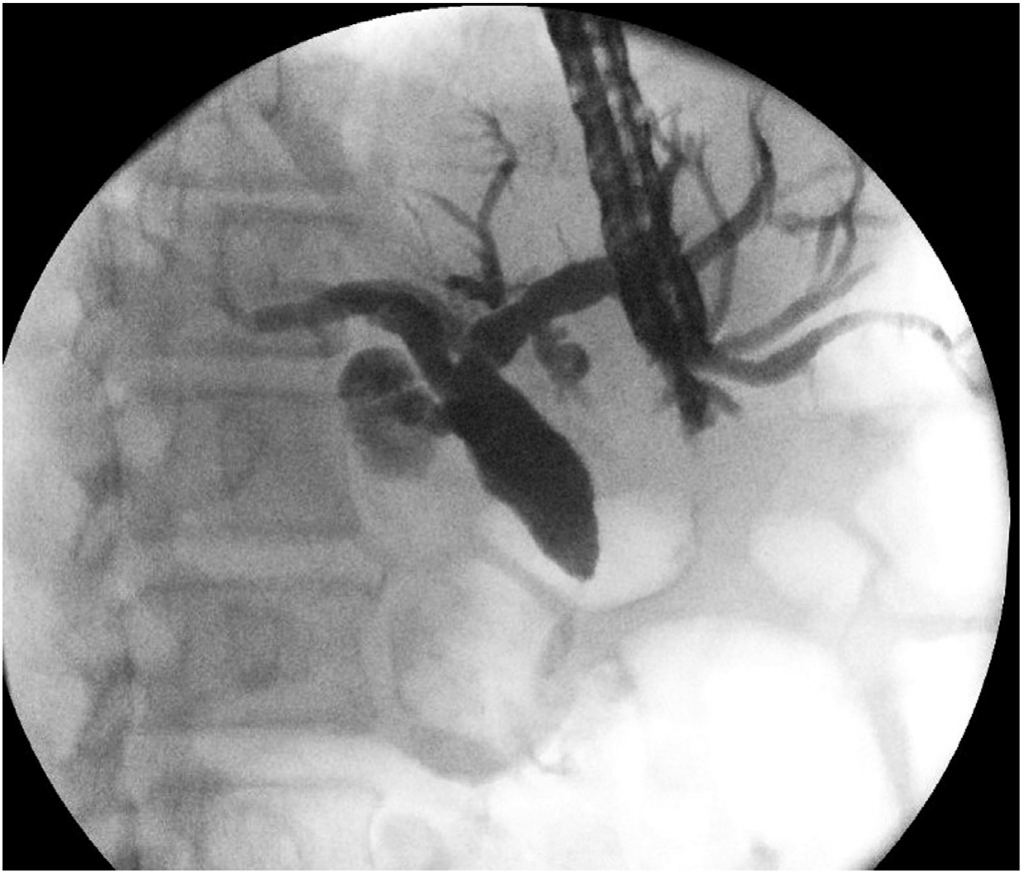
EUS-guided cholangiogram demonstrating a distal common bile duct stricture.

EUS could not obtain a good-quality image of the head of the pancreas through the gastric pouch. Due to the clinical suspicion of pancreatic malignancy, an EUS-guided cholangiogram was obtained. It revealed a distal common bile duct stricture with a dilated biliary tree upstream (Fig. 1). Hence, we decided to deploy a lumen-apposing metal stent (LAMS) connecting the remainder of the stomach to the proximal jejunum to enable retrograde EUS imaging of the head of the pancreas.

**Figure 2:**
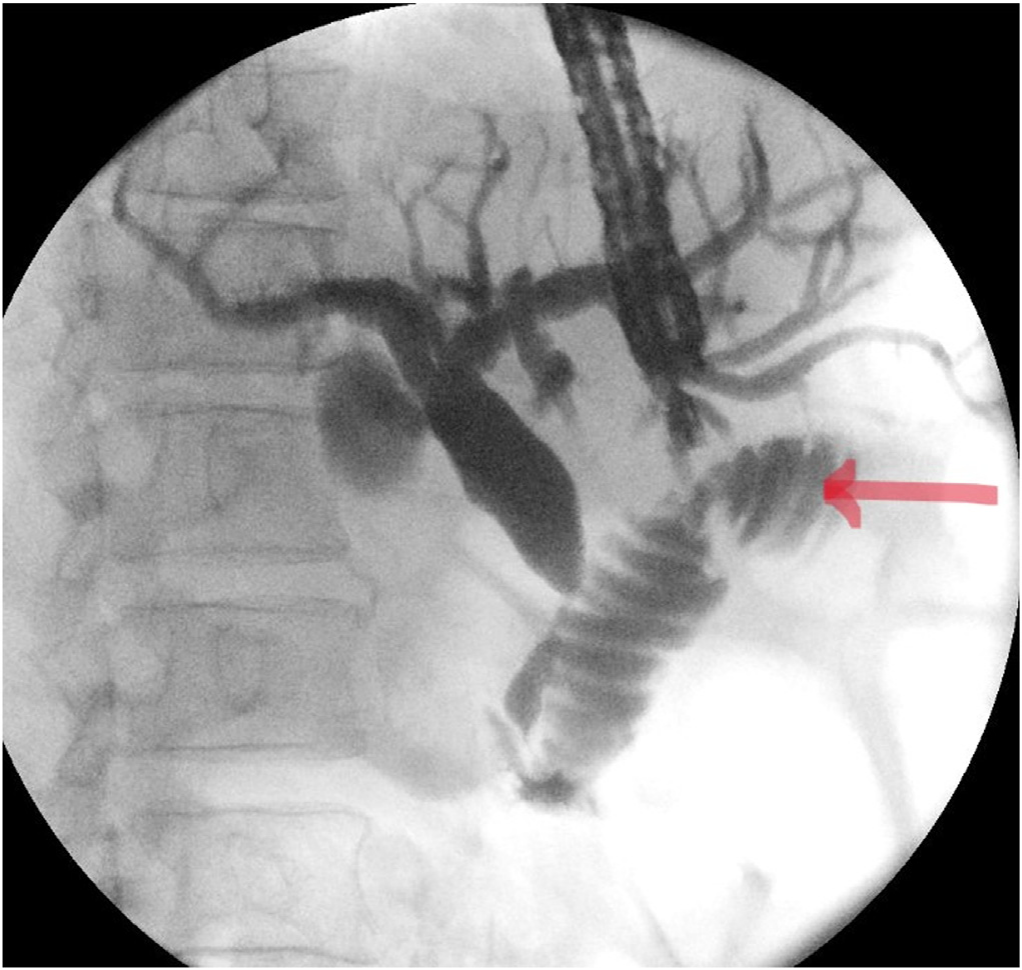
EUS-guided cholangiogram resulted in opacifying the proximal jejunum with contrast.

**Figure 3:**
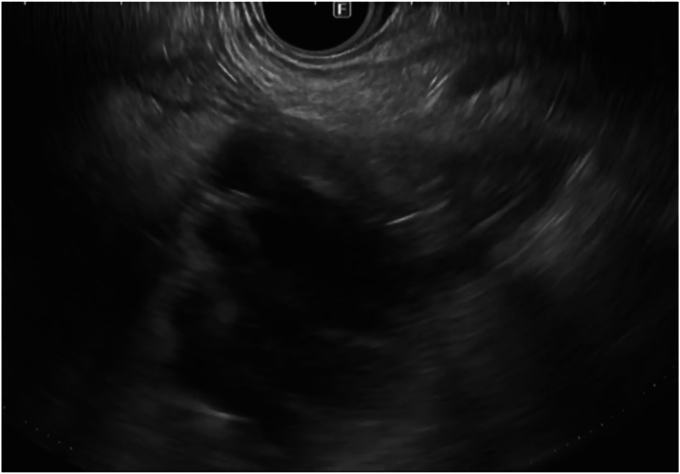
EUS image of the proximal jejunal loop after EUS-guided cholangiogram.

**Figure 4:**
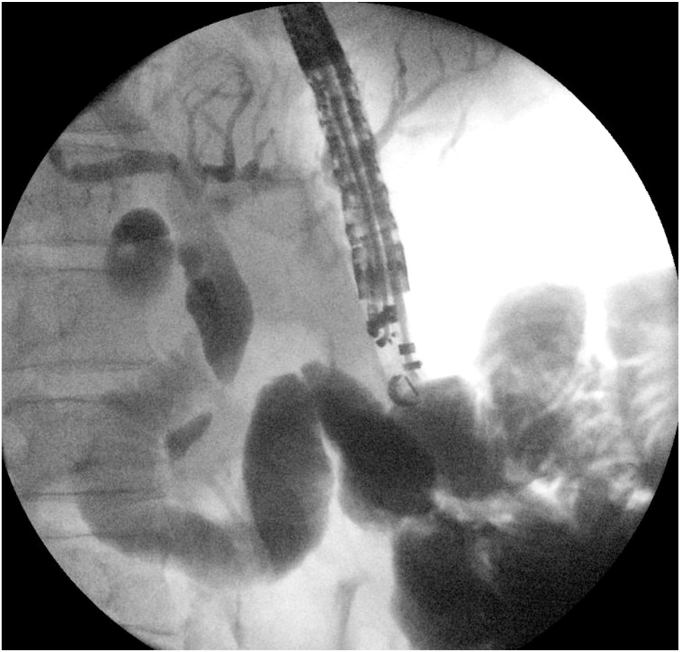
The proximal jejunum after further distention with contrast via a direct needle puncture technique.

**Figure 5:**
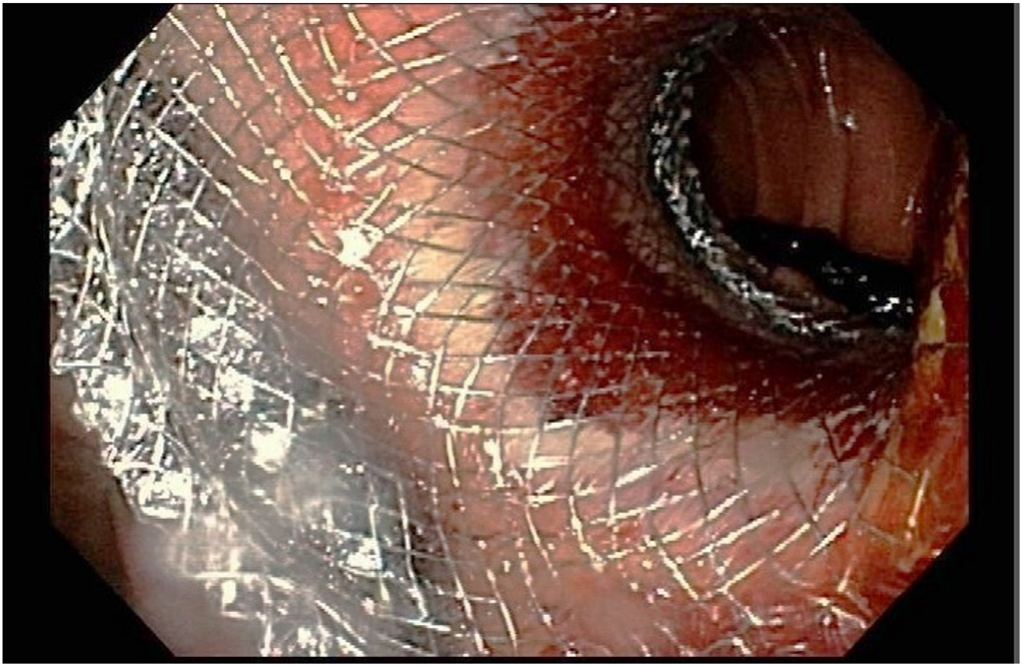
A view of the endoscopic gastrojejunostomy after dilating lumen-apposing metal stent.

The proximal jejunum was partially opacified after the cholangiogram (Fig. 2) and was identified on EUS (Fig. 3). It was further distended with contrast using a direct puncture technique^3^ (Fig. 4). A freehand technique was used, and an electrocautery-enhanced LAMS (2 cm) was deployed and dilated up to 2 cm (Fig. 5). CT confirmed a gastroenterotomy located just distal to the ligament of Treitz. This location allows the echoendoscope (125-cm working length) to reach the proximal duodenum.

Given the risk of perforation associated with stent dislodgement and the lack of urgency, we elected to allow the track to mature before imaging the head of the pancreas with EUS. A temporary internal-external percutaneous drain was inserted to avoid cholangitis.

Although in our experience a 2-week interval is sufficient for the track to mature, the second-step procedure was done 4 weeks later due to scheduling and patient-related factors.

A gastroscope was entered through the LAMS and was advanced retrograde to the duodenum. A wire was left to guide the echoendoscope as the gastroscope was withdrawn.

**Figure 6:**
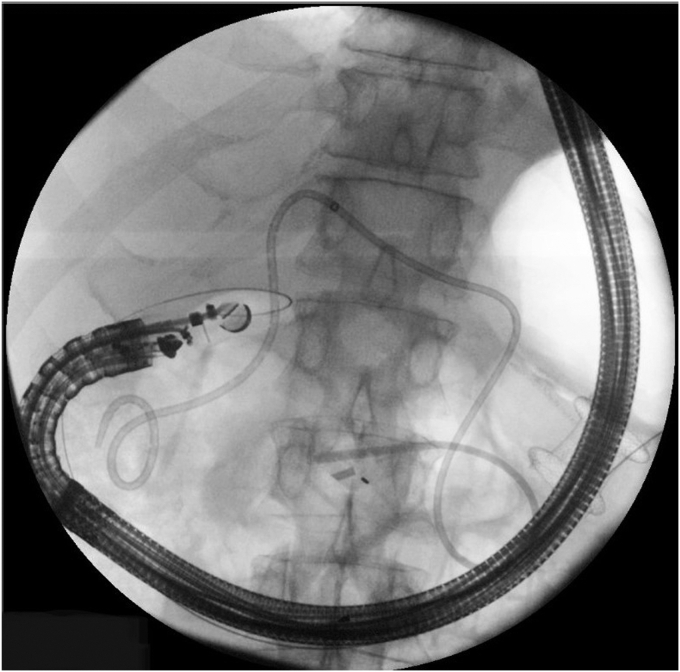
A fluoroscopic image of the echoendoscope through lumen-apposing metal stent reaching the proximal duodenum.

**Figure 7:**
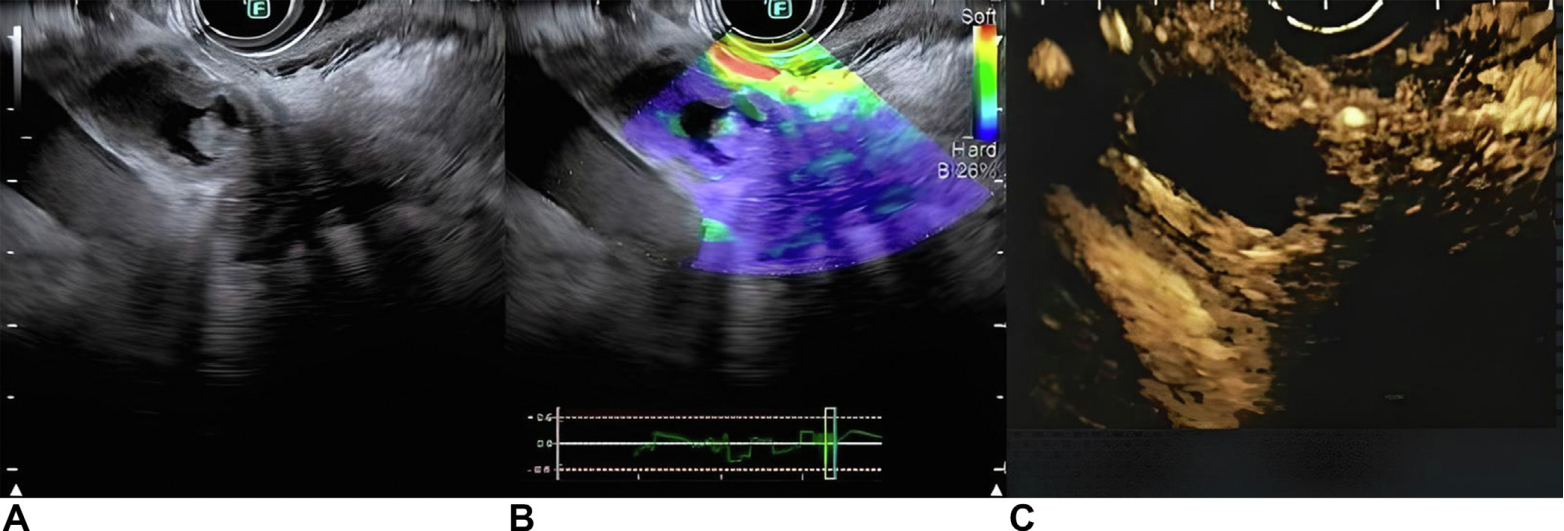
(**A**) EUS image of the mass at the head of the pancreas. (**B**) The mass appeared predominantly blue on elastography, consistent with hard tissue. (**C**) The mass was hypoenhancing after contrast injection.

A linear echoendoscope was advanced through the same route guided by the wire to the proximal duodenum (Fig. 6). US imaging of the head of the pancreas showed a hypoechoic mass measuring 3 cm. The mass was predominantly blue on elastography^4^ and hypo-enhancing after contrast injection^5^ (Fig. 7). These imaging features are suspicious for pancreatic cancer. There were no liver metastases or vascular invasion.

**Figure 8:**
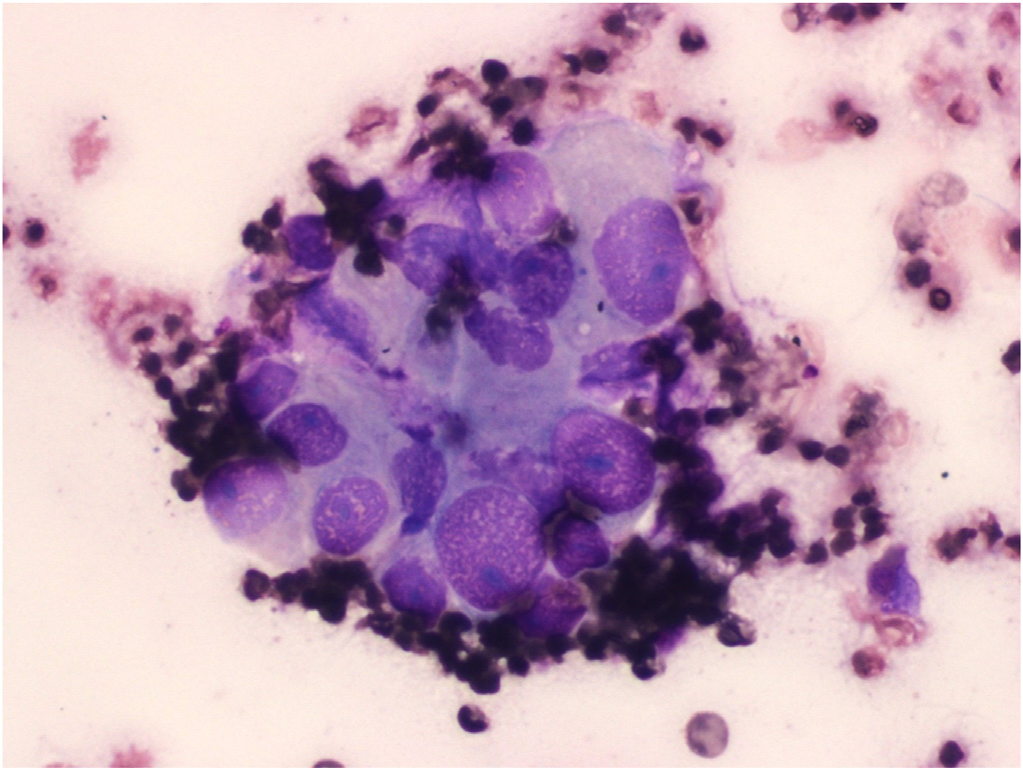
Fine-needle biopsy (Diff-Quik staining at 400 × magnification) revealing cancer cells.

Fine-needle biopsy with on-site pathology evaluation was consistent with adenocarcinoma (Fig. 8).

**Figure 9:**
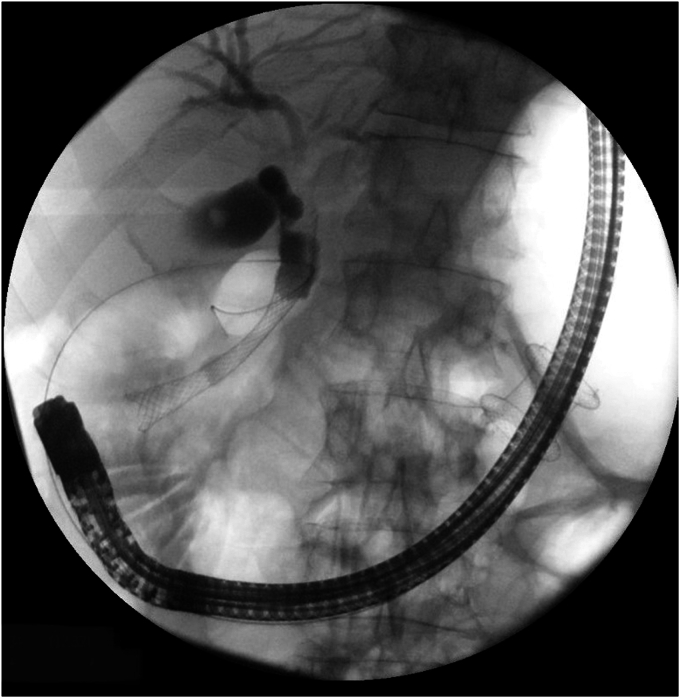
Fluoroscopic image of a fully covered metal stent across the biliary stricture.

Finally, a rendezvous ERCP was performed replacing the percutaneous drain with a fully covered metal stent (Fig. 9).

There were no adverse events. He was referred for oncological evaluation and was treated with neoadjuvant chemotherapy followed by a Whipple procedure.

## Conclusions

1. EUS with fine-needle biopsy has become the standard of care for establishing the diagnosis of pancreatic cancer due to its high diagnostic yield and accuracy.
2. Selected altered anatomies present a challenge to image the head of the pancreas with EUS due to the inability of the echoendoscope to reach the duodenum.
3. In this presentation, we demonstrated the feasibility of using EUS in a patient with distal gastrectomy and Roux-en-Y reconstruction to establish an early diagnosis of imaging-occult cancer at the head of the pancreas.
4. A direct needle puncture technique can facilitate EUS-guided gastroenterotomy when positioning a catheter to distend the targeted jejunal loop is not feasible (Video 1, available online at www.videogie.org).

## Disclosure

The authors disclosed no financial relationships relevant to this publication.

## Supplementary data

Video 1 Retrograde endosonography for diagnosing imaging-occult cancer at the head of the pancreas in a patient with distal gastrectomy and Roux-en-Y reconstruction.
